# Bilateral pulmonary cavitary mucinous adenocarcinoma in an immunocompetent patient: a case report and literature review

**DOI:** 10.1186/s12890-025-03989-z

**Published:** 2025-11-18

**Authors:** Xiao Meng, Rong He, Wei Zhang

**Affiliations:** Department of Pulmonary and Critical Care Medicine, Affiliated Hospital of Shandong University of Traditional Chinese Medicine, Jinan, China

**Keywords:** Pulmonary mucinous adenocarcinoma, Cavitary lesion, Computed tomography, Atypical presentation

## Abstract

We report an unusual case of pulmonary mucinous adenocarcinoma (PMA) in a 67-year-old immunocompetent male who presented with atypical imaging features that initially resulted in misdiagnosis. Chest CT revealed bilateral diffuse patchy opacities and consolidation with multiple cavities and nodules, mimicking infectious diseases. After unsuccessful anti-infective treatment, CT-guided lung biopsy confirmed PMA. The presence of multiple cavitary changes represents a rare manifestation of PMA that differs significantly from its characteristic imaging appearance, posing substantial diagnostic challenges. This case highlights the importance of maintaining a high index of clinical suspicion for PMA, even when imaging features are atypical, as such presentations can lead to delayed diagnosis and inappropriate treatment.

## Introduction

PMA is a distinct subtype of non-small cell lung cancer, accounting for approximately 3–10% of lung adenocarcinomas [1]. Its pathological characteristics include tumor cells secreting large amounts of extracellular mucin, forming mucin lakes or glandular structures [2]. Imaging manifestations often lack specificity and are easily confused with pulmonary infections [3]. Typically, PMA imaging manifestations consist of nodules or consolidation with ground-glass opacity, often accompanied by mucoid impaction signs, whereas cavitation in PMA is relatively uncommon, increasing diagnostic difficulty [4].

Imaging examination plays a key role in the diagnosis and differential diagnosis of PMA. Chest CT, as the preferred examination method, has typical manifestations that help suggest the diagnosis, but atypical imaging features often lead to clinical misdiagnosis as infectious conditions (such as pneumonia or lung abscess) or other types of lung cancer [5, 6]. We report a case of PMA manifesting primarily as bilateral multiple cavities. Through detailed analysis of its imaging features and differences from typical manifestations, combined with a review of the literature, we sought to provide a reference for clinicians and improve recognition of atypical imaging manifestations of this disease.

## Case report

Patient: Male, 67 years old, farmer. In January 2025, he developed a cough and copious white mucous sputum with chest tightness and dyspnea following a cold. He was treated at a local township hospital with intravenous ceftriaxone for 10 days, concurrent oral azithromycin for 5 days, and nebulized budesonide combined with salbutamol. The patient showed mild symptomatic improvement, but symptoms worsened again within 1–2 weeks after discontinuation of treatment. The patient’s recurrent symptoms despite adequate antibiotic therapy with appropriate spectrum and duration suggested possible non-infectious etiologies, though this was not further investigated at that time. He subsequently visited a county-level hospital where chest CT showed “bilateral pulmonary inflammation with multiple nodules,” and he was referred to our hospital on March 26, 2025. His medical history included a 30-year history of coronary heart disease and a smoking history of 30 years (20 cigarettes/day), with no history of immunodeficiency or long-term corticosteroid use.

**Fig. 1:**
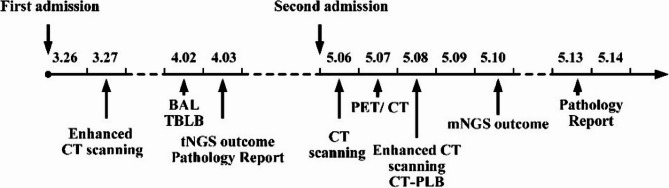
Timeline of diagnostic procedures and interventions from initial presentation through final diagnosis

Physical examination upon admission revealed: temperature, 36.7 °C; pulse, 60 beats/min; respiration, 20 breaths/min; blood pressure, 158/97mmHg; SpO₂, 94% (without oxygen). Bilateral lung sounds were coarse, with moist rales audible in the left lung. The rest of the physical examination showed no obvious abnormalities. The sequence of key clinical events following admission is summarized in the timeline (Fig. 1).

Laboratory investigations revealed the following: Complete blood count showed white blood cells 11.36 × 10⁹/L (reference value 3.5–9.5 × 10⁹/L), neutrophils 6.49 × 10⁹/L (reference value 1.8–6.3 × 10⁹/L), C-reactive protein (CRP) 3.80 mg/L (reference value 0–10.00.00 mg/L), procalcitonin (PCT) < 0.05ng/ml. Tumor marker analysis revealed cytokeratin 19 fragment antigen 21 − 1 (CYFRA21-1) 17.17ng/ml (reference value 0–3.3ng/ml), neuron-specific enolase (NSE) 20.34ng/ml (reference value 0–16.3ng/ml), carcinoembryonic antigen (CEA) 8.29ng/ml (reference value < 5ng/ml), and squamous cell carcinoma (SCC) 2.920ng/ml (reference value 0–2.7ng/ml). (1,3)-β-D-glucan assay (G test), galactomannan assay (GM test), and T-cell-based interferon-gamma release assay for tuberculosis (IGRA) were all negative.

**Fig. 2:**
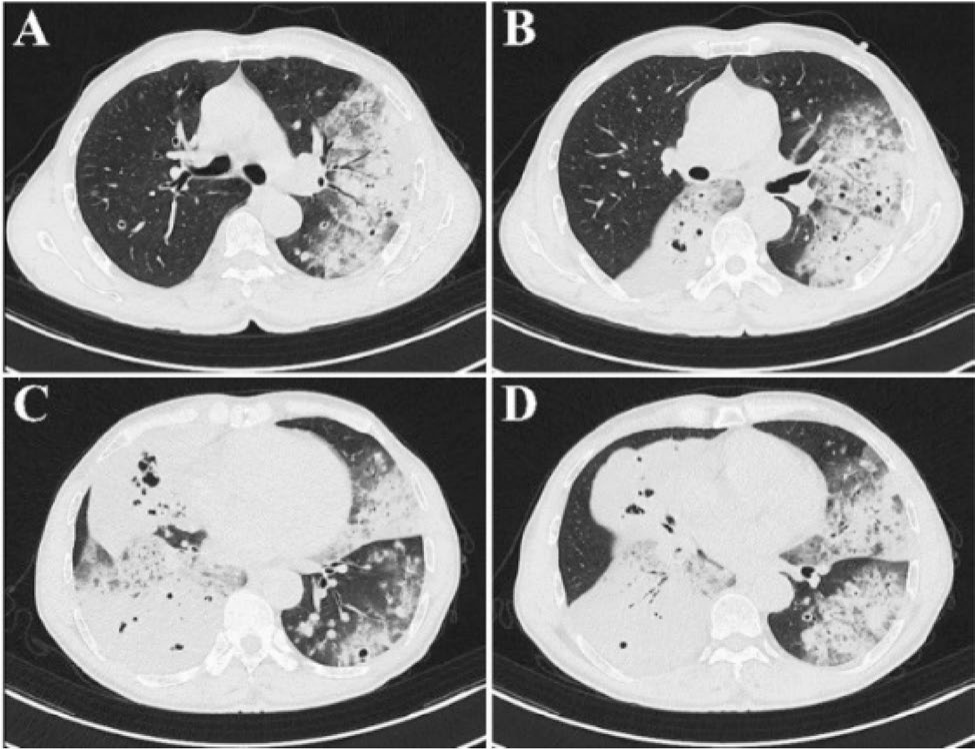
Contrast-enhanced CT images (A-D) during first admission demonstrate bilateral diffuse patchy opacities and consolidation with multiple cavities, predominantly affecting the right middle-lower lobe and left lung, with associated nodules

**Fig. 3:**
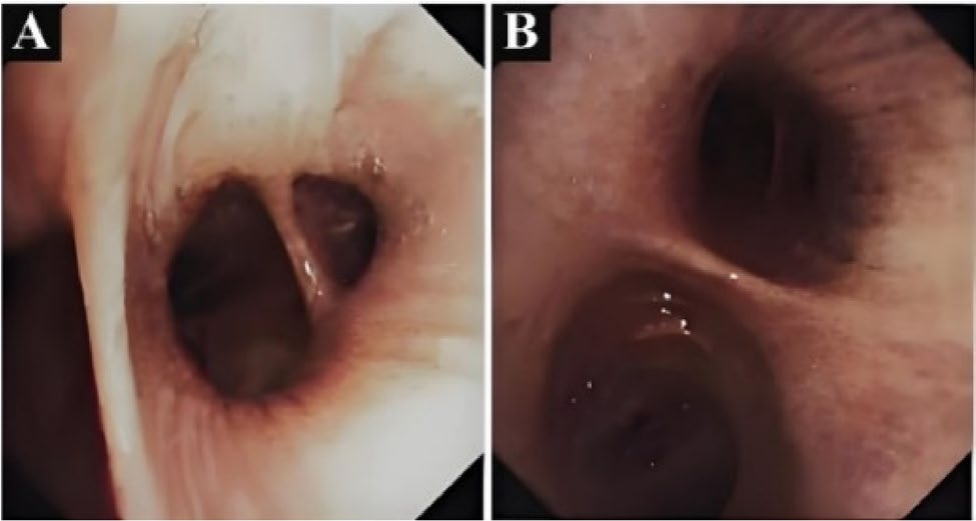
Bronchoscopic examination of the right lower lobe basal segment showing mucosal congestion with preserved luminal patency. **A** Left lower lobe bronchus. **B** Right lower lobe bronchus

**Fig. 4:**
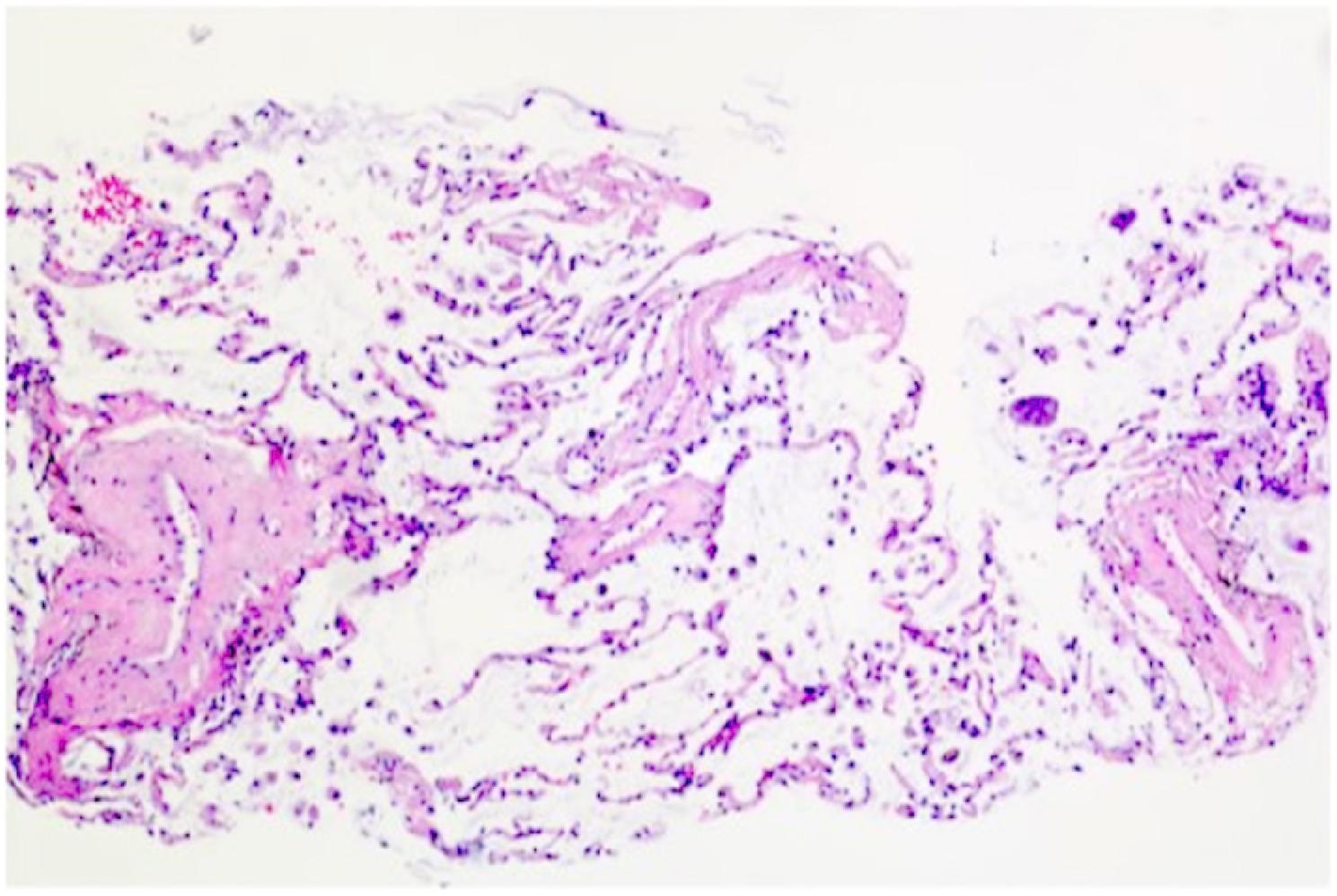
Initial lung biopsy (H&E staining) showing alveolar septa without significant thickening, scattered histiocytes in alveolar spaces, and minimal mucin-producing cells with mild atypia in the alveolar walls

Contrast-enhanced chest CT demonstrated bilateral diffuse patchy opacities and consolidation with multiple cavities, prominent in the right middle-lower lobe and left lung. Both lungs also showed multiple nodules of varying sizes, some ring-shaped with cavitary changes inside, with a maximum diameter of approximately 1.2 cm. The right lower lobe bronchus showed patchy opacities with compromised distal luminal patency (Fig. 2). Bronchoscopy showed congestion of the right lower lobe basal segment bronchial mucosa (Fig. 3). Bronchoalveolar lavage fluid targeted next-generation sequencing (tNGS) detected SARS-CoV-2 and influenza virus H1N1, with no bacterial or fungal sequences found. Cytological examination revealed inflammatory cells with no atypical cells identified. Histopathological examination of the lung biopsy specimen revealed focal proliferation of mucinous cells in the alveolar wall with mild atypia and low cell count (Fig. 4). Initial diagnosis was “pulmonary infection,” treated with levofloxacin, mezlocillin sodium, and other anti-infective and symptomatic treatments. The patient’s symptoms improved slightly, and he was discharged.

**Fig. 5:**
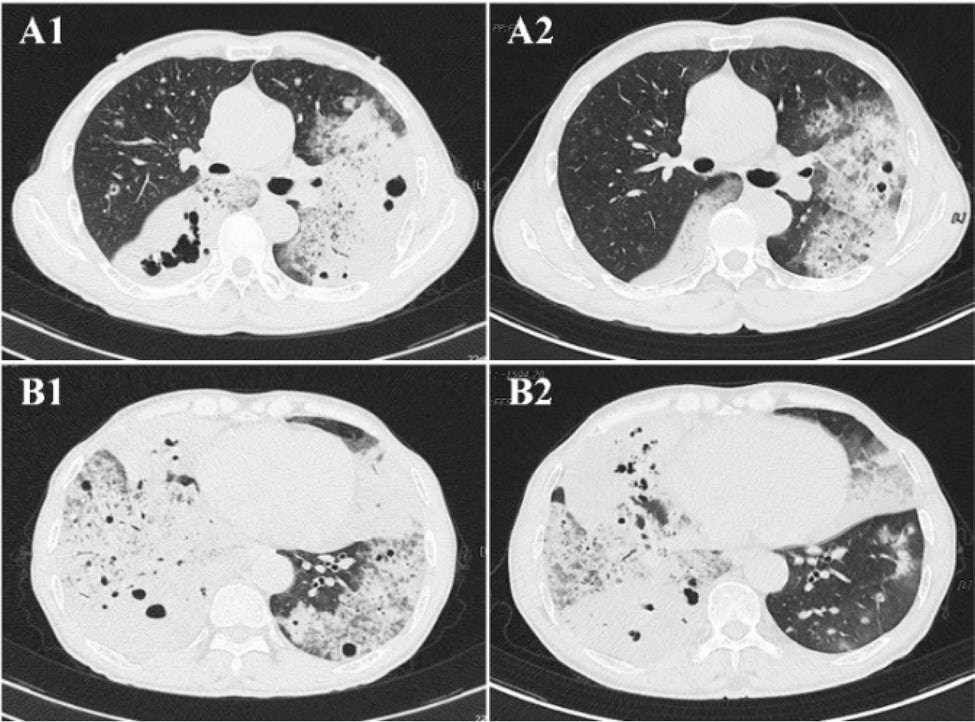
Serial chest CT comparison showing disease progression between first (**A2**, **B2**) and second admission (**A1**, **B1**), with increased extent of bilateral lung lesions, greater number of cavities, and enlargement of nodules

**Fig. 6:**
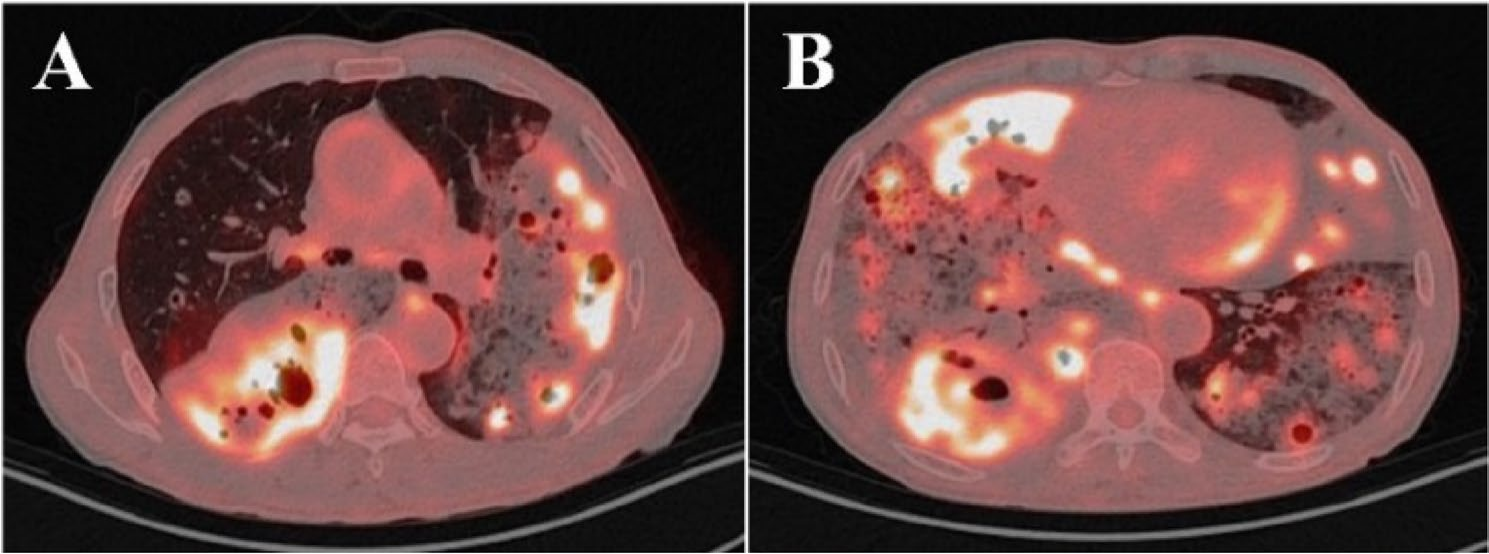
PET/CT images (A, B) revealed multiple hypermetabolic patchy consolidations and nodules with cavitation in both lungs (SUVmax 10.0)

**Fig. 7:**
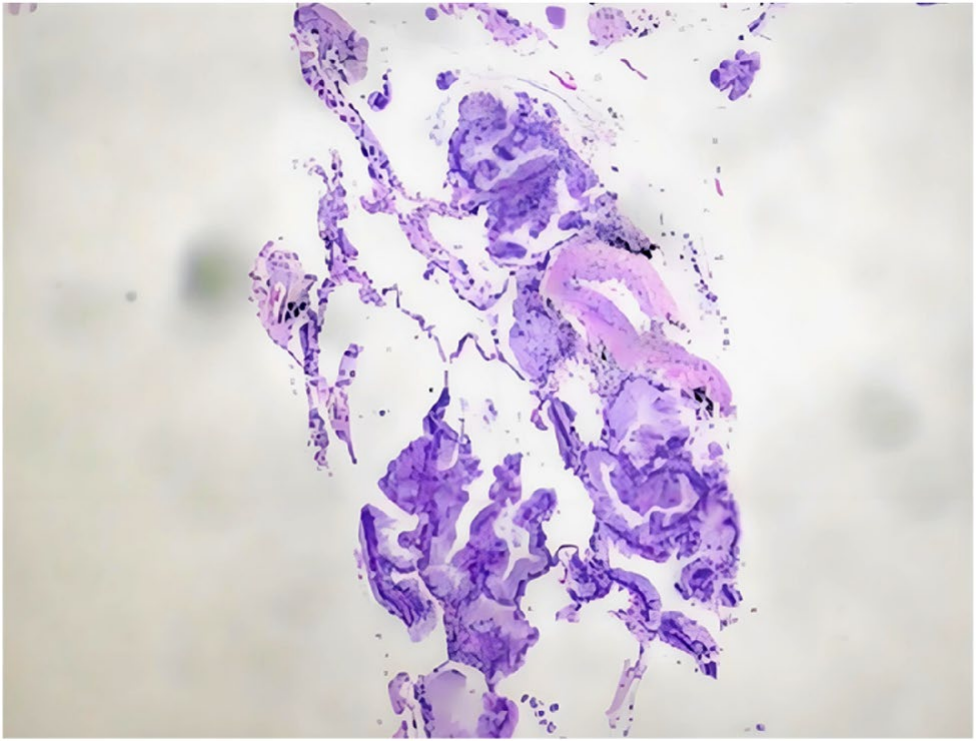
Definitive pathological diagnosis (H&E staining) demonstrating atypical mucin-secreting glandular structures consistent with pulmonary mucinous adenocarcinoma. Immunohistochemistry: CK7(+), TTF-1(-), Ki-67(15%+), P53(wild-type)

On May 6, 2025, the patient was readmitted due to symptomatic worsening. Follow-up chest CT showed increased extent of bilateral lung lesions compared to the previous examination, an increased number of cavities, and some nodules enlarged to 1.3 cm in diameter (Fig. 5). Given the patient’s progressive clinical deterioration despite adequate antibiotic therapy, elevated tumor markers (CYFRA21-1, NSE, CEA, SCC), and the concerning pattern of enlarging cavities, PET/CT was performed to assess the metabolic activity of the lesions and to provide additional information for guiding further diagnostic steps. Positron emission tomography/computed tomography (PET/CT) showed multiple bilateral hypermetabolic areas with a SUVmax of 10.0 (Fig. 6). Although the high metabolic activity was initially interpreted as potentially indicative of an infectious process, we acknowledged that such findings could also be consistent with malignancy, as both conditions can demonstrate significant FDG uptake. The persistent clinical deterioration and imaging progression, combined with elevated tumor markers, prompted us to proceed with a more definitive tissue sampling procedure via CT-guided percutaneous lung biopsy. On May 8, 2025, the patient underwent additional contrast-enhanced CT and CT-guided percutaneous lung biopsy. Lung tissue specimen metagenomic next-generation sequencing (mNGS) showed a small amount of Aspergillus sequences (sequence number 1, relative abundance 24%), which was deemed likely due to background contamination during the procedure; therefore, fungal infection was excluded based on clinical correlation. Pathological examination revealed atypical mucinous cells arranged in glandular structures, with abundant mucin-rich cytoplasm. Immunohistochemistry showed CK7 (+), TTF-1 (-), Ki-67 (15%+), P53 (wild type), consistent with a pulmonary mucinous adenocarcinoma diagnosis (Fig. 7). After diagnosis, chemotherapy with pemetrexed and cisplatin combined with anlotinib targeted therapy was initiated. Subsequently, the patient continued regular chemotherapy at the local hospital. The patient remains in good overall condition during follow-up.

## Discussion and literature review

As a distinct subtype of adenocarcinoma, PMA’s CT manifestations are closely related to the pathological characteristics of tumor mucin secretion. Pneumonia-like patchy opacities or large areas of consolidation are commonly observed, with often poorly defined boundaries, showing infiltrative growth and forming halo signs or reverse halo signs [7]. The mucoid bronchial sign in the consolidation area presents typical tree-in-bud changes, with bronchial dilation accompanied by intraluminal mucus plugs, and bronchial wall stiffness and truncation. Post-contrast images demonstrate no enhancement. This triad—comprising bronchial dilation, mucus plugging, and absence of wall enhancement—exhibits high specificity [8]. Some cases may present as single or multiple nodules or masses. Single lesions are rounded with smooth edges, and internal nodular mucin retention can form bubble signs. Multiple lesions present as randomly distributed snowflake signs in both lungs, some with punctate calcification caused by calcium salt deposition in mucin [9].

This case’s CT manifestations differ significantly from typical PMA, showing bilateral diffuse patchy opacities and consolidation with multiple cavities and nodules, similar to common bacterial pneumonia, organizing pneumonia, pulmonary fungal infection (such as Aspergillus), or pulmonary lymphoma, which can readily lead to clinical misdiagnosis [10]. Cavities are common in various lung diseases, including pneumonia, lung abscess, tuberculosis, lung squamous cell carcinoma, and pulmonary metastases, but are relatively rare in pulmonary mucinous adenocarcinoma. Research indicates that cavitary lesions in PMA patients occur in only 3–10% of cases. The presence of cavitation in PMA carries significant prognostic implications that warrant special attention. As demonstrated in previous studies, patients with cavitary PMA have markedly worse outcomes compared to their non-cavitary counterparts, with 5-year survival rates dropping to 22% versus 48% for non-cavitary patients [11]. This survival disparity may be attributed to several factors: First, cavitation often signifies a more aggressive tumor biology characterized by increased mucin production and tissue destruction; it is frequently associated with more advanced disease stages at diagnosis; and the cavitary pattern may represent a more invasive phenotype with increased metastatic potential. Furthermore, the atypical imaging appearance of cavitary PMA, as exemplified by our case, often leads to diagnostic delays during which the disease progresses, potentially contributing to the poorer prognosis. These findings underscore the critical importance of maintaining high clinical suspicion for malignancy in patients presenting with cavitary lung lesions, particularly when infectious etiologies have been adequately excluded.

A literature search was conducted using PubMed and other databases with the keywords “Pulmonary mucinous adenocarcinoma,” “CT features,” and “cavitation.” Relevant literature published within the last 10 years was selected to summarize characteristics of CT manifestations of PMA and compare them with this case (Table 1).

**Table 1:** Comparative analysis of typical CT manifestations of pulmonary mucinous adenocarcinoma and this case

Feature	Literature-reported Typical Manifestations	Manifestations in this case	Difference analysis
Main Imaging Type	Nodular opacities or ground-glass opacities with consolidation	Bilateral diffuse patches and consolidation, multiple cavities and nodules	This may be related to tumor heterogeneity and extensive pulmonary involvement, leading to more complex and atypical imaging manifestations
Nodule Characteristics	Single or multiple nodules, rounded, smooth edges	Bilateral multiple nodules, some ring-shaped with cavitary changes inside, blurred edges	Nodules may represent invasive tumor growth, with bubble lucencies suggesting early cavitation or residual mucin in alveolar spaces
Cavity Formation	Rare, incidence3%−10%	Multiple bilateral cavities	This may be related to tumor cells secreting large amounts of mucin, causing local tissue necrosis and liquefaction, as well as intratumoral pressure changes, forming cavities. This difference suggests the tumor in this case may have higher mucin secretion activity and tissue destructiveness
Bronchial Changes	Tree-in-bud changes with bronchial dilation, mucus plugs, and no wall enhancement(triad)	Right lower lobe bronchus shows patchy consolidation with compromised distal luminal patency	May be due to different tumor growth patterns or mucin distribution differences, making them less recognizable on imaging
Other Signs	Bubble sign, snowflake sign, punctate calcification visible	No typical bubble sign or punctate calcification, mixed presence of cavities and nodules	This may be related to tumor histological characteristics and disease stage, suggesting the tumor microenvironment may favor necrosis or cavity formation mechanisms (such as mucin discharge, tumor ischemia)

In this case, due to atypical CT manifestations and bronchoscopic lung biopsy pathology showing only mild atypical mucinous cell proliferation in the alveolar wall with low cell count, no clear evidence for PMA diagnosis was found. Finally, PMA was diagnosed through CT-guided lung puncture biopsy. This case offers an important clinical insight: in patients diagnosed with “pneumonia” who exhibit a poor response to anti-infective therapy or experience recurrent symptoms, the possibility of malignancy should be considered, especially when CT shows mixed cavities and nodules, requiring dynamic observation of imaging changes.

Regarding the role of PET/CT in this diagnostic workup, although it can sensitively detect metabolically active lesions, its utility in distinguishing between malignant and infectious processes remains limited, particularly before obtaining definitive tissue diagnosis [12]. In this case, the high SUV values (SUVmax 10.0) demonstrated significant metabolic activity but could not reliably distinguish between active infection and malignancy, as both conditions can exhibit similar FDG uptake patterns. The decision to perform PET/CT was based on the need to assess disease extent and metabolic activity in the context of progressive symptoms and elevated tumor markers; however, the ultimate diagnostic determination relied on histopathological confirmation rather than metabolic imaging findings alone.

As with other lung cancers, histopathological examination remains the gold standard for diagnosing PMA. In clinical practice, bronchoscopic lung biopsy is a commonly employed method for obtaining pathological samples; however, due to constraints such as limited operating space, diagnostic yield may be suboptimal owing to inadequate sampling. CT-guided lung puncture biopsy can improve diagnostic yield, especially for peripheral lesions [13].

Treatment of pulmonary mucinous adenocarcinoma should follow individualized comprehensive treatment principles based on tumor staging, molecular characteristics, and pathological subtypes [14]. Early-stage disease is managed with surgical radical treatment; locally advanced inoperable disease is treated with concurrent chemoradiotherapy combined with immunotherapy consolidation; and advanced disease management emphasizes molecular typing-driven precision strategies [15]. This patient was diagnosed with advanced-stage PMA. In the absence of further testing for gene mutations or PD-L1 expression, a regimen of cisplatin, pemetrexed, and anlotinib was administered for anti-angiogenic therapy [16].

The clinical implications of this case extend beyond its rarity and emphasize fundamental principles of diagnostic vigilance. When patients present with pneumonia-like lesions that demonstrate poor response to appropriate antibiotic therapy, especially in the setting of progressive symptoms, enlarging lesions, or elevated tumor markers, clinicians should promptly consider malignancy and pursue tissue diagnosis. The threshold for pursuing invasive diagnostic procedures should be lower in such scenarios, as early histopathological confirmation can prevent prolonged inappropriate treatment and ensure timely initiation of appropriate oncological management. This case reinforces that atypical imaging presentations should not deter clinicians from pursuing definitive tissue diagnosis when clinical suspicion for malignancy exists.

## Conclusion

This case reports a pulmonary mucinous adenocarcinoma in an immunocompetent patient manifesting primarily as multiple bilateral cavities, with CT features significantly different from typical PMA. Clinicians should recognize that PMA can present with atypical imaging features. For pulmonary cavities with poor anti-infective treatment effects, the possibility of PMA should be considered, and timely pathological biopsy should be performed for definitive diagnosis to avoid misdiagnosis.

## Data Availability

The data that support the findings of this study are available from the corresponding author upon reasonable request.

## Abbreviations

PMA: Pulmonary mucinous adenocarcinoma
CT: Computed tomography
CRP: Procalcitonin
CYFRA21-1: Cytokeratin 19 fragment antigen 21-1
CEA: Carcinoembryonic antigen
NSE: Neuron-specific enolase
SCC: Squamous cell carcinoma
IGRA: Interferon-gamma release assay
G test: (1,3)-β-D-glucan test
GM test: Galactomannan test
tNGS: Targeted next-generation sequencing
PET/CT: Positron emission tomography/Computed tomography
mNGS: Metagenomic next-generation sequencing
